# A Bayesian network meta-analysis on the efficacy and safety of eighteen targeted drugs or drug combinations for pulmonary arterial hypertension

**DOI:** 10.1080/10717544.2018.1523257

**Published:** 2018-11-15

**Authors:** Sumei Wang, Miao Yu, Xiangchun Zheng, Shangjuan Dong

**Affiliations:** a Department of Emergency, Dongfang Hospital Beijing University of Chinese Medicine, Beijing, China;; b Department of Emergency, Beijing University of Chinese Medicine Third Affiliated Hospital, Beijing, China;; c Department Respiration, Dongfang Hospital Beijing University of Chinese Medicine, Beijing, China

**Keywords:** Pulmonary arterial hypertension, 6 minutes walking distance, functional class, randomized controlled trial, network meta-analysis

## Abstract

Pulmonary arterial hypertension (PAH) can be relieved by pharmacological interventions, especially the targeted drug, which is classified into endothelin receptor antagonist, phosphodiesterase 5 inhibitor, prostaglandin I_2_, soluble guanylate cyclase stimulator and selective non-prostanoid prostacyclin receptor agonist. To solve the contradictions existing in reported trials and provide a comprehensive guideline for clinical practice. PubMed, Embase, Cochrane library, and clinicaltrials.gov were searched. The basic information about the article, trial, arm, intervention, and the detailed data of outcome, including 6 minutes walking distance (6MWD) change, WHO functional class (FC) improvement, Borg dyspnea score (BDS) change, cardiac index (CI) change, mean pulmonary arterial pressure (mPAP) change, mean right arterial pressure (mRAP) change, pulmonary vascular resistance (PVR) change, clinical worsening, hospitalization, death, severe adverse events (SAEs), and withdrawal were extracted. The rank of treatments was estimated. 10,230 cases provided the firsthand comparison data about targeted drugs for treating PAH. For 6MWD, ambrisentan + tadalafil, vardenafil, and sildenafil + bosentan were better than others. Epoprostenol, macitentan, and sildenafil represented a greater WHO FC improvement. Vardenafil and treprostinil were better for BDS. So were bosentan + epoprostenol and bosentan alone for CI. Iloprost plus bosentan, bosentan + epoprostenol, and epoprostenol were better for mPAP. Iloprost plus bosentan, bosentan alone, and selexipag could reduce PVR. Sildenafil, epoprostenol, and vardenafil had the highest probability to reduce the incidence of death and withdrawal. To conclude, vardenafil and iloprost + bosentan showed relatively better performance in both efficacy and safety. However, the therapeutic choice should be made according to both the feature of each therapy and the individual condition.

## Introduction

The average pressure of pulmonary artery (PAP) equal or greater than 25 mmHg detected via right heart catheterization under the quiescent condition at the sea level is the gold standard of pulmonary hypertension diagnosis (Simonneau et al., 2004; Cottin et al., 2010). According to the World Health Organization (WHO) classification, the disease along with hemodynamic characteristics, pulmonary capillary wedge pressure (PCWP) less than 15 mmHg and pulmonary vascular resistance (PVR) no less than 3 Wood units, belongs to Group I, pulmonary arterial hypertension (PAH) (Farber & Loscalzo, 2004). Electrocardiogram, chest radiography, magnetic resonance imaging, cardiopulmonary exercise testing, and other general examinations can also help to diagnose PAH (Galie et al., 2009b). However, this disease can be idiopathic, hereditable, or accompanied by other situation (Prins & Thenappan, 2016). The cause of it varies from congenital heart disease to HIV infection, from medication to pulmonary capillary hemangiomatosis, which leads to several treatment strategies targeting to distinct pathogeny (Simonneau et al., 2004). Although the exact global value of PAH is unknown, the prevalence of PAH is ranged from 10.6 to 26.0 cases per million adult inhabitants across parts of Europe and the United States (McGoon et al., 2013). Most of the patients suffer from fatigue, syncope, dyspnea, angina, hemoptysis, and even right heart failure (Rubin, 1997). A mean survival time of PAH was 14.9 ± 0.8 years, but for idiopathic PAH, it was only 2.8 ± 0.9 years (D'Alonzo, 1991; Ogawa et al., 2014).

PAH is a progressive hemodynamic and pathophysiological condition, which cannot be cured so far but can only be alleviated. Supportive therapy and referral strategy are safe but with limited efficacy. While, surgical procedure, like atrial septostomy and lung transplantation, can be effective but invasive as well (Galie et al., 2013). For Group I PH, the application of vasoactive substances is broader with a compromise between efficacy and safety. Endothelin receptor antagonist (ERA), phosphodiesterase 5 inhibitor (PDE-5i), prostaglandin I_2_ (PGI2), soluble guanylate cyclase stimulator (sGCS), and selective non-prostanoid prostacyclin receptor agonist (sPRA) are common choices for PAH with diverse mechanism. ERA is a dual endothelin receptor antagonist, targeting to ET_A_ and ET_B_ at the same while, and its represents are bosentan, sitaxentan (Elliott et al., 1998). PDE-5i is a selective inhibitor of cGMP specific type 5 phosphodiesterase, and its first compound, sildenafil, was approved by food and drug administration (FDA) in 2005 (Duarte et al., 2013). In 2009, two sGCS drugs designed for intracellular NO receptor, cinaciguat and riociguat began their clinical trials (Lasker et al., 2011). Epoprostenol, a synthetic PGI2, is also an available treatment for PAH (Rubin, 1990). And another prostacyclin receptor targeted drug, sPRA, with higher selective came out in recent year (Simonneau et al., 2012).

Nonetheless, the existing problem is how to make an optimal choice in clinical practice. Although there were many trials and systematic review providing plenty of precious experience and helpful suggestions, some potential conflicts due to error, poor quality, and other restrictions, make the situation more complex. In 2006, both Hoeper et. al. and McLaughlin et. al. evaluated the benefit of inhaled iloprost combined with bosentan over bosentan alone, however, they went to divergence (Hoeper et al., 2006a, McLaughlin et al., 2006). In Hoeper’s trial, the mean changes of 6 minutes walking distance (6MWD) were 1 ± 27 m (*p* = .84) and −9 ± 100 m (*p* = .65) for the control group and the combination group respectively with placebo-corrected difference −10 m (*p* = .49), which meant no positive effect was showed. While an increased exercise capacity was manifested in the treated group with the change 30 ± 60 m (*p* = .001) over 4 ± 61 m (*p* = .69) of the control group and placebo-corrected difference 26 m (*p* = .051). Several network meta-analysis was published recently. However, the existing network meta-analysis compared the efficacy of different drug categories but not specific drugs (PMID: 28507431, 27615023, 29128622) and the included endpoints were relatively limited.

Therefore, our goal was to perform a comprehensive network meta-analysis is acute to update former ones with high-quality randomized controlled trials (RCTs), more interventions and more endpoints involved, to solve the existed contradictions and provide a more convincing guideline for clinical practice of PAH.

## Materials and methods

### Identification strategy

Available RCTs were identified from the Internet database, including PubMed, Embase, Cochrane Library and clinicaltrials.gov, with searching terms including the disease ‘pulmonary arterial hypertension’, the interventions ‘endothelin receptor antagonist’, ‘phosphodiesterase 5 inhibitor’, ‘prostaglandin I_2_’, ‘soluble guanylate cyclase stimulator’, ‘selective non-prostanoid prostacyclin receptor agonist’, and concrete name of drugs, the type of literature ‘randomized controlled trial’, ‘meta-analysis’, and their synonyms were used jointly. Besides, every mentioned trial in each meta-analysis or systematic review was also retrieved manually. Preferred Reporting Items for Systematic Reviews and Network Meta-Analyses (PRISMA) was strictly followed during this systematic review and network meta-analysis study reporting [PMID: 26030634].

### Inclusion and exclusion criteria

The trial reported by each article could be selected as the data source after it passed through the examination, in light of the following inclusion and exclusion criteria. As to the inclusion criteria, (i) it must be an RCT, without limitation on blinding; (ii) the subjects researched by the trial must be diagnosed with PAH (WHO Group I PH) and no extra confinement on its causes; (iii) the compared interventions must be a targeted drug or drug combination for PAH or placebo, without requirement on specific target or mechanism; (iv) at least one measurable comparison outcome must be mentioned. While, for the exclusion criteria, (i) the length of follow-up must not be shorter than 8 weeks; (ii) the disease of subjects must not involve other types of PH, other than Group I; (iii) the trial investigating on the unreleased drug or drug which cannot be covered by the loop would be excluded; (iv) the trial which concentrated on the assessment of dosage or mode of administration would be removed. The final list was determined by two reviewers individually, and any disagreement would be solved by a panel discussion.

### Data extraction and endpoint

For each trial disclosed by the included articles, the basic information about the article, including author and published year; about the trial, including blinding, etiology of PAH, study period and gender proportion; about the study arm, including mean age and sample size; about the intervention, including drug, dosage, method of drug administration; and outcomes were summarized for further analysis. 6MWD, WHO functional class (FC), Borg dyspnea score (BDS), cardiac index (CI), mean pulmonary artery pressure (mPAP), mean right arterial pressure (mRAP), pulmonary vascular resistance (PVR), clinical worsening, hospitalization, death, severe adverse event (SAE), and withdrawal were taken as endpoints.

6MWD, as a functional exercise capacity measure, is generally deemed as the primary efficacy index for PAH treatment with characteristics of harmlessness, economic effectiveness, accessibility and reproducibility (Guyatt et al., 1985). Lower score indicates a worse function. For healthy adults, the distance covered in 6 minutes is between 400 m and 700 m (Casanova et al., 2011). WHO FC is a subjective evaluation marker of cardiopulmonary function with four levels, in terms of patient’s daily activity and the fourth class is the worst condition (Taichman et al., 2009), while BDS is a noninvasive indicator to assess the function of respiratory muscle with value ranging from 6 to 20 (von Leupoldt et al., 2006). As to CI, it is the quotient of cardiac output divided by body surface area, a good predictor of heart performance with a normal range 2.6–4.2 L/min/m^2^ at rest (Aessopos et al., 1995). mPAP is a direct diagnostic standard of PAH with a norm 9–18 mmHg (Kovacs et al., 2009). Another good indicator, PVR, is the drag force must be overcome to create a flow in the pulmonary circulation with a reference 0.25–1.6 Wood units (Reddy et al., 2015). mRAP reflects the volume of reinfusion and the capacity of pumping of the heart through measuring the blood pressure in the right atrium with a norm 2–6 mmHg (Paradis et al., 1989). Besides, some discrete variables, like clinical worsening, the aggregate of cases including censoring due to poor improvement or worsening, hospitalization, lung transplantation, death, and any other deteriorate situation, are also useful outcomes to evaluate the efficacy of the treatment for PAH as well (Rubin et al., 2002; Galie 2006,). And the incidence of withdrawal and SAEs were recorded to assess the safety of intervention. The detailed data extraction methods used for 6MWD change were specified in Supplementary Material 1.

### Statistical analytic method

Among all twelve endpoints, six continuous and four discrete variables were related to the evaluation of efficacy, and the other two discrete ones concerned with the aspect of safety. Seven efficacy-related endpoints were extracted as change or improvement, which is the difference between baseline and value at the terminal, with standard deviation (SD). While for the most discrete variables, only the number of case at the end of study was recorded. During the course of statistical analysis, the continuous variable was treated as mean difference (MD) and the discrete one was calculated as odds ratio (OR). Their 95% CrI was also estimated to show the significance. The interval containing 0 for MD and 1 for OR predicts no significant difference.

A traditional meta-analysis was performed at the first to test the heterogeneity of the fixed-effects model through Cochran’s Q methods and I squared statistic. Referring to the *p*-value less than .05 or I squared statistic over 50%, a significant heterogeneity was identified and the random-effects model would be applied in the further analysis. Then the indirect data was obtained from the primary evidence. After pooling them together, a network meta-analysis was done. All these progress was completed with the help of software R.

Network graph was plotted to demonstrate the providers of direct comparison by nodes and their connection by lines. The size of node and the width of line are proportional to the total sample size and the number of supported trials, respectively. The network analysis results were display in the slash table altogether and the forest plot with key comparisons. Surface under the cumulative ranking curve (SUCRA) for each endpoint was estimated in the table to rank the interventions. Moreover, node-splitting figure was used to exhibit the inconsistency between direct and indirect evidence, and heat plot explained this inconsistency with more details and show the contribution of direct data to the network estimate.

### Subgroup analysis

As sitaxsentan was withdrawn from the markets for hepatic damage in 2010, we conducted a subgroup analysis excluding sitaxsentan for network comparison of drugs in use on the market.

## Results

### Literature identification

**Figure 1.**
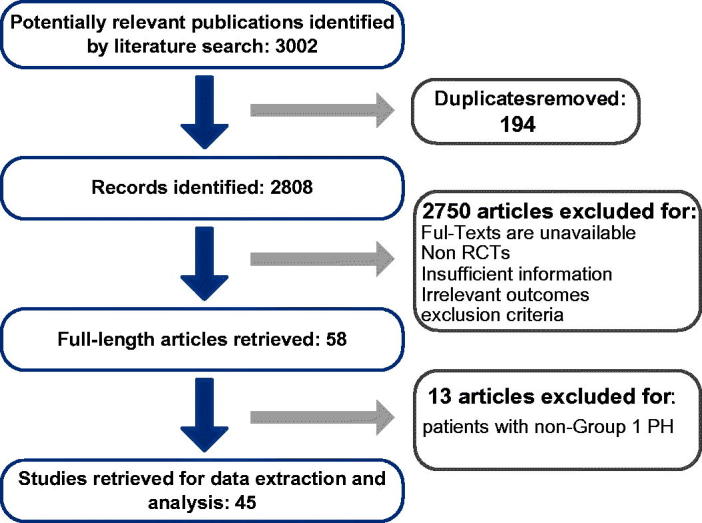
Flowchart for the process of screening out the included studies.

As shown in Figure 1, according to the identification strategy mentioned in the methods, 3002 publications were identified through an electronic database. Full manuscripts of 194 articles were retrieved after removing the duplicates and screening title and abstract. And 58 articles were included as they met the inclusion criterion. Among them, according to exclusion criteria, another 13 trials involving patients with non-Group 1 PH were excluded. Eventually data from 45 trials were retrieved as primary evidence for further analysis (Rubin, 1990; Barst et al., 1996; Badesch, 2000; Channick et al., 2001; Badesch et al., 2002; Galie et al., 2002; Rubin et al., 2002; Barst et al., 2003; McLaughlin et al., 2003; Barst et al., 2004; Humbert et al., 2004; Oudiz et al., 2004; Galie et al., 2005; Wilkins et al., 2005; Barst et al., 2006; Galie, 2006; McLaughlin et al., 2006; Hoeper et al., 2006b; Badesch et al., 2007; Simonneau, 2008; Galie et al., 2008a; 2008b; 2009a; Hiremath et al., 2010; Iversen et al., 2010; McLaughlin et al., 2010; Jing et al., 2011; Sandoval et al., 2012; Simonneau et al., 2012; Tapson et al., 2012; Ghofrani et al., 2013; Jing et al., 2013; Pulido et al., 2013; Tapson et al., 2013; Zhuang et al., 2014; Chin et al., 2015; Hoendermis et al., 2015; McLaughlin et al., 2015; Rosenkranz et al., 2015; Rubin et al., 2015; Sitbon et al., 2015; Webb et al., 2015; Galie et al., 2015a; 2015b; Vizza et al., 2017). Characteristics of included trials.

**Figure 2.**
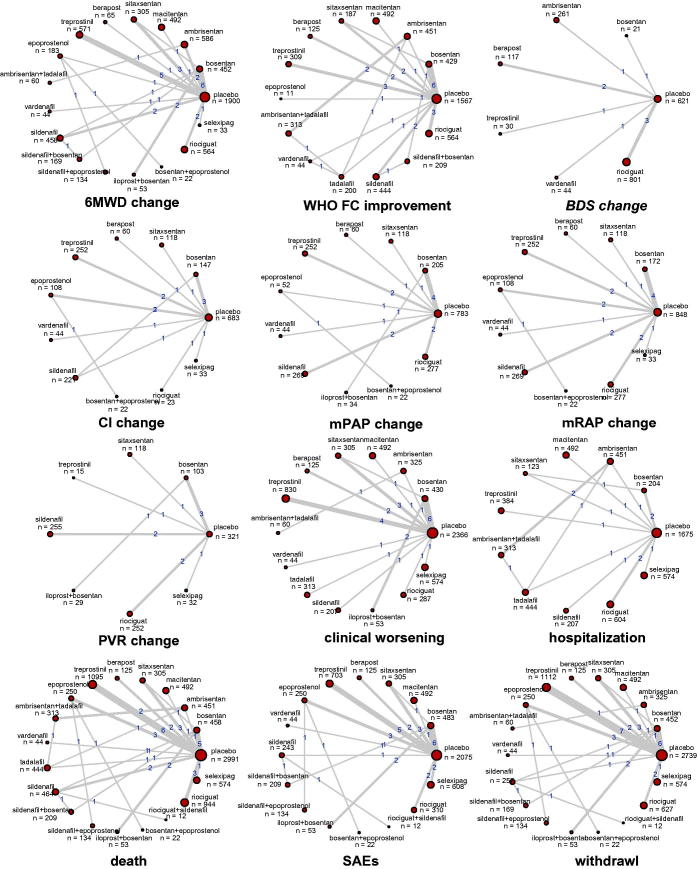
Network structure for all outcomes. The network plots show direct comparison of different treatments, with node size corresponding to the sample size. The number of included studies for specific direct comparison decides the thickness of solid lines.

In this network meta-analysis, a total of 10,230 cases were included from 45 qualified trials, published from April 1990 to September 2017 and 40 of RCTs were double-blinded. The etiology of the subjects covered the idiopathic, heritable and PAH relating to other factors like connective tissue disease, HIV, drug use or toxin exposure. More details of main characteristics for each trial were listed in Table S1. The direct comparisons gathered from 45 trials were illustrated in Figure 2, in terms of twelve different endpoints. The pair of bosentan, an ERA intervention, and placebo, and the pair of treprostinil, a PGI-2 treatment, and placebo was the most contributive ones with large pile of articles supporting.

### 6 Minutes walking distance, 6MWD

**Figure 3.**
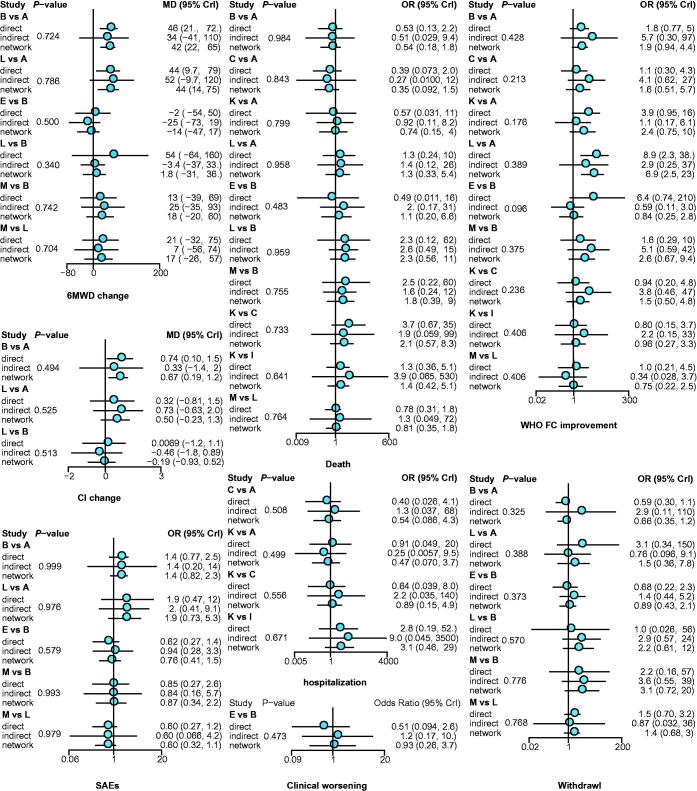
Node-splitting results for outcomes. *p* < .05 indicates inconsistency between direct and indirect evidence. A: placebo; B: bosentan; C: ambrisentan; D: macitentan; E: sitaxsentan; F: berapost; G: treprostinil; H: epoprostenol; I: ambrisentan + tadalafil; J: vardenafil; K: tadalafil; L: sildenafil; M: sildenafil + bosentan; N: sildenafil + epoprostenol; O: iloprost + bosentan; P: bosentan + epoprostenol; Q: riociguat + sildenafil; R: riociguat; S: selexipag.

6MWD is a common indicator to assess the efficacy of treatments for PAH. In this analysis, nine interventions showed their significant benefits over placebo, containing the combination of ambrisentan and tadalafil (MD: 79, 95% CrI 15–140), vardenafil (MD: 69, 95% CrI 23–120), the combination of sildenafil and bosentan (MD: 62, 95% CrI 21–100), the combination of iloprost and bosentan (MD: 57, 95% CrI 7.6–110), epoprostenol (MD: 47, 95% CrI 0.36–94), sildenafil (MD: 45, 95% CrI 17–76), bosentan (MD: 43, 95% CrI 22–64), ambrisentan (MD: 43, 95% CrI 8.5–77), and treprostinil (MD: 33, 95% CrI 11–56), which agreed with the SUCRA rank in Table 1 that the combination of ambrisentan and tadalafil (0.8513) was the first, vardenafil (0.8172) and the combination of sildenafil and bosentan (0.7703) were the following, and no obvious inconsistency was illustrated in Figure 3.

**Table 1.** Surface under the cumulative ranking curve (SUCRA) results for outcomes.

Treatment	6MWD change	WHOFC improvement	BDS change	CI change	mPAP change	mRAP change	PVR change	Clinical worsening	Hospitalization	Death	SAEs	Withdrawal
Placebo	0.0950	0.1247	0.0524	0.1601	0.0792	0.2807	0.2001	0.1298	0.2215	0.3068	0.5331	0.4537
Bosentan	0.5731	0.4197	**0.5903**	**0.7154**	**0.6424**	**0.7139**	**0.8170**	0.6550	**0.5396**	0.5518	0.3437	0.6639
Ambrisentan	0.5679	0.3266	0.5031	–	–	–	–	0.6130	0.4268	0.6676	–	0.6603
Macitentan	0.3047	**0.8385**	–	–	–	–	–	0.3159	0.4533	0.2015	**0.7147**	0.5359
Sitaxsentan	0.3808	0.3909	–	0.4171	0.4455	0.5105	0.0139	**0.6706**	**0.7511**	0.4933	0.5162	**0.6994**
Berapost	0.3678	0.2442	0.3283	0.2196	0.1880	0.4511	–	0.4688	–	0.4928	**0.8179**	0.1360
Treprostinil	0.4376	0.5017	**0.8771**	0.4501	0.1198	0.4494	0.2346	0.2369	0.2237	0.4417	**0.6964**	0.2723
Epoprostenol	0.6204	**0.9667**	–	**0.6105**	**0.6862**	**0.6160**	–	–	–	**0.7404**	0.1614	0.3889
Ambrisentan + tadalafil	**0.8513**	0.5071	–	–	–	–	–	**0.8467**	**0.8411**	0.5404	–	0.5432
Vardenafil	**0.8172**	0.3100	**0.8802**	0.4892	0.5631	0.5595	–	**0.8312**	–	**0.8405**	0.6851	**0.9813**
Tadalafil	–	0.4990	–	–	–	–	–	0.3646	0.5097	0.4045	–	–
Sildenafil	0.5991	**0.7901**	–	0.5518	0.4654	0.4360	0.4664	0.4411	**0.6587**	0.2514	0.2311	0.3664
Sildenafil + bosentan	**0.7703**	**0.7008**	–	–	–	–	–	–	–	0.3453	0.4775	0.2482
Sildenafil + epoprostenol	0.3194	–	–	–	–	–	–	–	–	**0.9712**	0.3505	**0.6923**
Iloprost + bosentan	**0.7098**	–	–	–	**0.9490**	–	**0.9931**	**0.9034**	–	**0.7201**	0.5958	**0.6885**
Bosentan + epoprostenol	0.3651	–	–	**0.9900**	**0.9338**	**0.7909**	–	–	–	0.4084	0.3396	0.2066
Riociguat + sildenafil	–	–	–	–	–	–	–	–	–	0.4603	0.1951	0.3393
Riciguat	0.3183	0.3799	0.2687	0.3375	0.4275	**0.5838**	**0.5608**	0.1910	0.4845	0.4999	0.6490	0.5026
Selexipag	0.4022	–	–	**0.5588**	–	0.1083	**0.7141**	0.3320	0.3899	0.1623	**0.6929**	0.6212

6MWD: 6 minutes walking distance; FC: functional class; BDS: Borg dyspnea score; CI: cardiac index; mPAP: mean pulmonary artery pressure; mRAP: mean right arterial pressure; PVR: pulmonary vascular resistance; SAEs: severe adverse event. The bolded value is top three SUCRA under each endpoints.

### WHO functional class, FC

**Figure 4.**
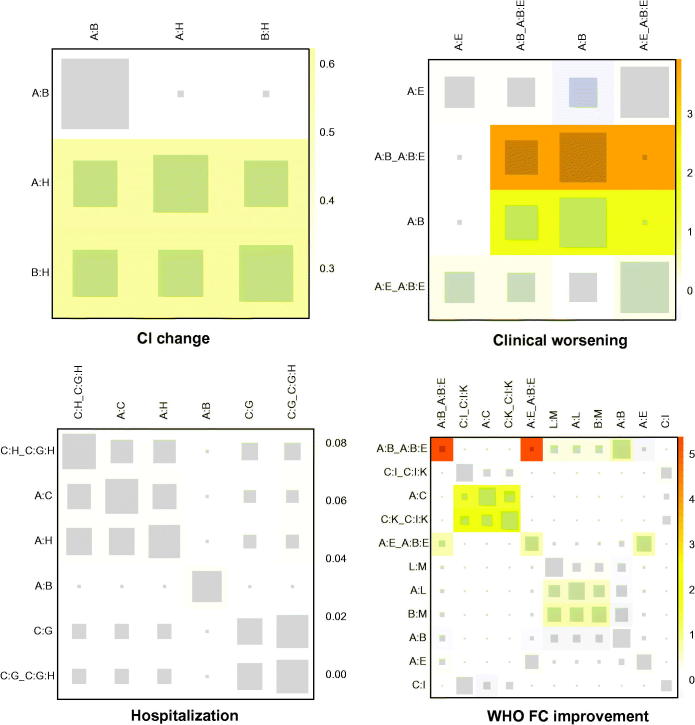
Heat plots of CI change, clinical worsening, hospitalization and WHO FC improvement. The area of the gray squares displays the contribution of the direct estimate in the design shown in the column to the network estimate in the design shown in the row. The colors are associated with the change in inconsistency between direct and indirect evidence. Blue colors indicate a decrease and warm colors indicate an increase (the stronger the intensity of the color, the stronger the change). A: placebo; B: bosentan; C: ambrisentan; D: macitentan; E: sitaxsentan; F: berapost; G: treprostinil; H: epoprostenol; I: ambrisentan + tadalafil; J: vardenafil; K: tadalafil; L: sildenafil; M: sildenafil + bosentan; N: sildenafil + epoprostenol; O: iloprost + bosentan; P: bosentan + epoprostenol; Q: riociguat + sildenafil; R: riociguat; S: selexipag.

WHO FC was a discrete variable used as the efficacy-related endpoint, and three treatments, epoprostenol (OR: 94, 95% CrI 4–5600), macitentan (OR: 12, 95% CrI 1.5–97), and sildenafil (OR: 7.1, 95% CrI 1.8–34), can improve this indicator distinctively in contrast to the placebo. And the SUCRA rank in Table 1 was epoprostenol (0.9667), macitentan (0.8385), and sildenafil (0.7901). Besides, epoprostenol also represented an extreme goodness over many other interventions, listed in Table S2. However, arresting inconsistency between direct and indirect evidence was observed in the comparison of bosentan and sitaxsentan (*p* = .0963), as shown in Figure 2, which was mainly derived from placebo, bosentan and sitaxsentan, suggested by heat plot in Figure 4.]

### Borg dyspnea score, BDS

As to BDS, an index for assessing the respiratory function of patients, only vardenafil (MD: −2.2, 95% CrI −3.9 to −0.51) and treprostinil (MD: −2.1, 95% CrI −3.4 to −0.79) were significantly better than placebo. Vardenafil (0.8802) and treprostinil (0.8771) were the first two tops, and bosentan (0.5903) was the third one, according to their SUCRA results in Table 1.

### Cardiac index, CI

**Figure 5.**
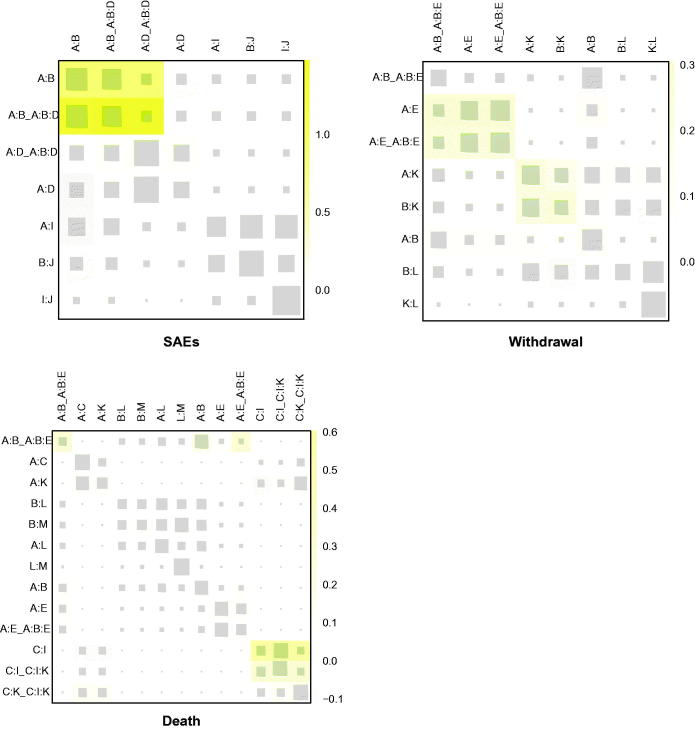
Heat plots for SAEs, withdrawal, and death.

To evaluate the heart function, CI was introduced. Bosentan plus epoprostenol (MD: 11, 95% CrI 2.6–20), as well as bosentan alone (MD: 0.67, 95% CrI 0.16–1.2) outperformed the placebo with statistical significance and bosentan plus epoprostenol was the optimal one with the best performance when compared to other interventions, inferred from Supplementary Table S2. Except for the combination of bosentan and epoprostenol (0.9900) and bosentan (0.7154), epoprostenol alone (0.6105) may also be a good alternation. No remarkable inconsistency was shown by node-splitting plot in Figure 5, but the yellow color in heat plot, among placebo, bosentan, and epoprostenol should be paid attention to.

### Mean pulmonary artery pressure, mPAP

In the aspect of lowering mPAP, iloprost plus bosentan (MD: −14, 95% CrI −20 to −8.4), bosentan plus epoprostenol (MD: −13, 95% CrI −20 to −6.8), epoprostenol (MD: −6.7, 95% CrI −11 to −2.5), bosentan (MD: −6, 95% CrI −8.4 to −3.7), sildenafil (MD: −4.2, 95% CrI −7.6 to −0.95), and riociguat (MD: −3.8, 95% CrI −7.6 to −0.14) were quite good. And their SUCRA values were iloprost plus bosentan (0.9490), bosentan plus epoprostenol (0.9338), and epoprostenol (0.6862). In addition to the superiority of the above two-drug combinations, the good performance of bosentan over berapost (MD: −4.96, 95% CrI −9.87 to −0.31) and treprostinil (MD: −5.67, 95% CrI −9.75 to −2.15), and the good performance of epoprostenol over treprostinil (MD: −6.39, 95% CrI −11.77 to −1.46) were testified as well, as shown in Table S2.

### Mean right arterial pressure, mRAP

Unlike the situation in reducing mPAP, no one manifested a significant advantage in regulating mRAP. On the contrary, selexipag (MD: 3.2, 95% CrI −3.5 to 9.8) even increased mRAP, though statistically insignificant, compared with placebo.

### Pulmonary vascular resistance, PVR

For PVR, the combination of iloprost and bosentan (MD: −680, 95% CrI −880 to −470), bosentan alone (MD: −430, 95% CrI −530 to −340), selexipag (MD: −350, 95% CrI −630 to −75), riociguat (MD: −210, 95% CrI −340 to −59), and sildenafil (MD: −150, 95% CrI −280 to −30) all exhibited their outstanding goodness, which was verified again by SUCRA rank in Table 1 that iloprost plus bosentan (0.9931), bosentan (0.8170), and selexipag (0.7141) were the top three. Regardless of these three, the benefits of riociguat (MD: −368.48, 95% CrI −574.32 to −142.26) and sildenafil (MD: −311.07, 95% CrI −515.25 to −104.93) over sitaxsentan were proved in Table S2 as well.

### Clinical worsening

Clinical worsening is the amount of any aggravations ever happened during the trial. The proportion of clinical worsening was significantly cut down in the group administrated with ambrisentan plus tadalafil (OR: 0.082, 95% CrI 0.0073–0.89), iloprost plus bosentan (OR: 0.060, 95% CrI 0.0051–0.36), sitaxsentan (OR: 0.24, 95% CrI 0.065–0.64), and bosentan (OR: 0.26, 95% CrI 0.077–0.53). Moreover, the combination of iloprost and bosentan was also better than riociguat (OR: 0.06, 95% CrI 0.00–0.57), treprostinil (OR: 0.08, 95% CrI 0.01–0.52), and macitentan (OR: 0.09, 95% CrI 0.00–0.80), and bosentan was better than treprostinil (OR: 0.32, 95% CrI 0.07–0.91), as illustrated in Table S2. However, referring to the SUCRA results in Table 1, iloprost plus bosentan (0.9034) had the highest probability to be the optimum in reducing the occurrence of clinical worsening, followed by ambrisentan plus tadalafil (0.8467) and vardenafil (0.8312). The inconsistency among placebo, bosentan, and sitaxsentan warned by the warm color in heat plot Figure 4 need to be noticed.

### Hospitalization

Hospitalization is another efficient endpoint for making comparison on efficacy, but no one demonstrated an obvious benefit. Sorted by the SUCRA value in Table 1, the top three in decreasing the case of hospitalization were ambrisentan and tadalafil (0.8411), sitaxsentan (0.7511), and sildenafil (0.6587). Furthermore, no distinguished inconsistency was observed.

### Death

As to the number of death, sildenafil combined with epoprostenol (OR: 0.023, 95% CrI 0.00062–0.23) and epoprostenol alone (OR: 0.28, 95% CrI 0.09–0.84) significantly reduce the incidence of death, compared with placebo. Besides, this combination (sildenafil + epoprostenol) was much better than many other treatments and epoprostenol demonstrated its good performance not only over placebo but also over macitentan (OR: 0.19, 95% CrI 0.03–0.93), which were detailed in the Table S2. Suggested by the SUCRA value in Table 1, the combination of sildenafil and epoprostenol (0.9712) was at the first place, vardenafil (0.8405) and epoprostenol (0.7404) were at the second and third, respectively. As demonstrated in Figure 3, the consistency of each pair was quite well with most values over 0.5.

### Severe adverse event, SAE

Because 95% CrI of the comparison embraced value 1, nearly no intervention was good enough to have a statistical significance, except for berapost, which could reduce the incidence of SAE in contrast to epoprostenol (OR: 0.23, 95% CrI 0.05–0.91) to a great extent. The safety of berapost (0.8179) was also supported by SUCRA results. Macitentan (0.7147) and treprostinil (0.6964) were next to it. As to the inconsistency, only slightly yellow squares among placebo, bosentan, and macitentan in heat plot Figure 5 may be concerned.

### Withdrawal

For withdrawal, taking placebo as the control, network analytic results described that vardenafil (OR: 0.045, 95% CrI 0.0011–0.33) could decrease its occurrence, but treprostinil (OR: 1.51, 95% CrI 1.01–2.16) might increase it. Additionally, the combination of vardenafil and treprostinil, sitaxsentan (OR: 0.19, 95% CrI 0.03–0.82) and selexipag (OR: 0.23, 95% CrI 0.03–0.92) expressed a superiority over berapost as well, as shown in Table S2. Except for vardenafil (0.9813), the SUCRA value of most interventions concentrated around 0.66. Among them, sitaxsentan (0.6994) and sildenafil plus epoprostenol (0.6923) were relatively outstanding. Furthermore, no informative inconsistency was observed.

### Subgroup analysis

SUCRA results of subgroup analysis after extracting sitaxsentan were shown in Table S3. Table S4 showed network meta-analysis results for all outcomes. The subgroup results of all treatments differed little from the global analysis.

## Discussion

On the basis of primary comparison data from 45 RCTs, the efficacy and safety of eighteen targeted drugs or drug combinations for PAH were analyzed, in terms of twelve aspects. The combination of iloprost and bosentan performed best for lowering mPAP, PVR, and decreasing the incidence of clinical worsening, while bosentan plus epoprostenol could improve CI and mRAP efficiently. Vardenafil was the medication with least withdrawal and the first choice to improve BDS. Ambrisentan plus tadalafil was significantly better than others on 6MWD and the occurrence of hospitalization. For improving WHO FC, reducing the incidence of death and decreasing SAEs, epoprostenol alone, sildenafil plus epoprostenol, and berapost revealed more beneficial than others, respectively. Unfortunately, no single therapy was outstanding in the majority of investigated endpoints. Relatively speaking, vardenafil and iloprost + bosentan showed a better performance in both efficacy and safety.

Most reported network meta-analysis about targeted treatments for PAH concentrated more on the comparison of category. Gao et. al. indicated the advantage of combination therapy in improving 6MWD (20.94 m, 95% CrI 6.94, 34.94, *p* = .003) and reducing mPAP (3.97 mmHg, 95% CrI −6.06, −1.88, *p* < .001) over PGI-2, which was consistent with our results (Gao et al., 2017). Then Ataru’s network meta-analysis study showed that bosentan and sildenafil used to improve 6MWD and WHOFC in PAH appeared to be more superior then ERA- and PDE5I-class drugs (Igarashi et al., 2016). In our analysis, for both endpoints, the drug combinations (ambrisentan + tadalafil and iloprost + bosentan) outperformed other treatments significantly. However, due to the variance between an identical category and the discrepancy of included trials, conflicts were unavoidable. Although both Jain et. al. and we agreed that PGI-2 had distinguished superiority on WHO FC (RR: 5.09, 95% CrI 2.32, 11.04) and SAEs (RR: 2.92, 95% CrI 1.68, 5.06), he considered riociguat, a sGSCs agent, was the optimal choice for decreasing clinical worsening (RR: 0.19, 95% CrI 0.05, 0.76), and ERA combined with PDE-5i (RR: 0.27, 95% CrI 0.14, 0.52) was next to it, which diverged with the results in this analysis. Our analysis showed that ambrisentan plus tadalafil, a combination of ERA and PDE-5i, were much better than many other treatments in terms of reducing clinical worsening, but riociguat nearly had no difference compared with placebo (Jain et al., 2017). Nonetheless, in another network meta-analysis conducted by Lin et. al., ERA plus PDE-5i (OR: 0.11, 95% CrI 0.02, 0.57) was deemed as the most effective one to reduce the clinical worsening, and no remarkable difference was observed between sGSCs and placebo, which was in accordance with our conclusion (Lin et al., 2018). As for oral treprostinil, Chin et al and Tapson et al (PMID: 26401252, 23669822) reported that oral treprostinil did not result in significant improvement in exercise capacity, which is consistent with our study for low ranking of treprostinil under 6MWD (0.4376), mRAP (0.4494) and mPAP (0.1198). As for oral drug vardenafil, which showed good performance both in efficacy and safety in our study, a double-blinded RCT of Jing et al in 2011 reported vardenafil is effective and well tolerated in patients with PAH (PMID: 21471085).

As the first network meta-analysis which directly evaluated the common specific targeted therapies, instead of the drug category, on a more comprehensive dimension with twelve aspects relating to efficacy or safety, it reduced the significant variance within drug groups. Although bosentan and sitaxsentan, both belonging to the ERAs, owned the same target, the former one was more effective in lowering PVR, while sitaxsentan demonstrated no superiority over placebo in terms of PVR change and was much less effective than bosentan (placebo vs. sitaxsentan, MD: 160, 95% CrI −9.7 to 320; bosentan vs. sitaxsentan, MD: −593.24, 95% CrI −783.18 to −401.25). This dilemma was not an exception, for withdrawal, two PDE-5i treatments, vardenafil and treprostinil acted out opposite as well (vardenafil vs. treprostinil, OR: 0.03, 95% CrI 0.01–0.23), under this tricky situation, grouping by individual drugs overweighed by the mechanism. However, the consequent problem was the inevitable error posed by the poor quality and limited size of included trials and small probability events. Therefore, more valuable data of RCTs should be included to update this analysis.

Differing from the existing network meta-analysis, this analysis provided a more straightforward clinical guideline, rather than proved the efficacy or safety of a typical target or mechanism. Even though Igarashi et. al., Zhang et. al., and Duo-Ji et.al. had made some attempts to investigate specific drugs, they were confined to oral medications or medications within the same category, such as prostacyclin analogs and ERAs (Igarashi et al., 2016; Zhang et al., 2016; Duo-Ji & Long, 2017). Meanwhile, these literatures offered another thought that it might be meaningful to make a subgroup analysis on dosage or drug delivery.

As a whole, this Bayesian meta-analysis suggested a rank with statistical significance for each endpoint. For majority of endpoints, the most beneficial treatments and their significance were concluded. To conclude, vardenafil and iloprost + bosentan showed relatively better performance in both efficacy and safety. However, the therapeutic choice should be made according to both the feature of each therapy and the individual condition.

## Data Availability

The datasets used and analyzed during the current study are available from the corresponding author on reasonable request.
